# Identification of Novel Autoantibodies Based on the Human Proteomic Chips and Evaluation of Their Performance in the Detection of Gastric Cancer

**DOI:** 10.3389/fonc.2021.637871

**Published:** 2021-02-26

**Authors:** Chi Cui, Yaru Duan, Cuipeng Qiu, Peng Wang, Guiying Sun, Hua Ye, Liping Dai, Zhuo Han, Chunhua Song, Kaijuan Wang, Jianxiang Shi, Jianying Zhang

**Affiliations:** ^1^BGI College & Henan Institute of Medical and Pharmaceutical Sciences, Zhengzhou University, Zhengzhou, China; ^2^Henan Key Laboratory of Tumor Epidemiology, Zhengzhou University, Zhengzhou, China; ^3^School of Basic Medical Sciences, Zhengzhou University, Zhengzhou, China; ^4^College of Public Health, Zhengzhou University, Zhengzhou, China; ^5^State Key Laboratory of Esophageal Cancer Prevention & Treatment, Zhengzhou University, Zhengzhou, China

**Keywords:** gastric cancer, proteomic chip, tumor-associated antigen (TAA), autoantibody, diagnostic model, immunodiagnosis

## Abstract

Autoantibodies against tumor-associated antigens (TAAbs) can be used as potential biomarkers in the detection of cancer. Our study aims to identify novel TAAbs for gastric cancer (GC) based on human proteomic chips and construct a diagnostic model to distinguish GC from healthy controls (HCs) based on serum TAAbs. The human proteomic chips were used to screen the candidate TAAbs. Enzyme-linked immunosorbent assay (ELISA) was used to verify and validate the titer of the candidate TAAbs in the verification cohort (80 GC cases and 80 HCs) and validation cohort (192 GC cases, 128 benign gastric disease cases, and 192 HCs), respectively. Then, the diagnostic model was established by Logistic regression analysis based on OD values of candidate autoantibodies with diagnostic value. Eleven candidate TAAbs were identified, including autoantibodies against INPP5A, F8, NRAS, MFGE8, PTP4A1, RRAS2, RGS4, RHOG, SRARP, RAC1, and TMEM243 by proteomic chips. The titer of autoantibodies against INPP5A, F8, NRAS, MFGE8, PTP4A1, and RRAS2 were significantly higher in GC cases while the titer of autoantibodies against RGS4, RHOG, SRARP, RAC1, and TMEM243 showed no difference in the verification group. Next, six potential TAAbs were validated in the validation cohort. The titer of autoantibodies against F8, NRAS, MFGE8, RRAS2, and PTP4A1 was significantly higher in GC cases. Finally, an optimal prediction model with four TAAbs (anti-NRAS, anti-MFGE8, anti-PTP4A1, and anti-RRAS2) showed an optimal diagnostic performance of GC with AUC of 0.87 in the training group and 0.83 in the testing group. The proteomic chip approach is a feasible method to identify TAAbs for the detection of cancer. Moreover, the panel consisting of anti-NRAS, anti-MFGE8, anti-PTP4A1, and anti-RRAS2 may be useful to distinguish GC cases from HCs.

## Introduction

Gastric cancer (GC) is one of the most important causes of cancer death in the world (1). It was reported that in 2018, there were 1,033,701 new cases of GC, accounting for 5.7% of total new cancer cases, ranking the fifth. The number of GC deaths was 782,685, accounting for 8.2%, ranking only after lung cancer and breast cancer (1). In China, the 5-year survival rate of GC patients is only around 10% because many patients are diagnosed at an advanced stage (2–4). The 5-year survival rate of early GC can be as high as 75% after surgery, radiotherapy, and chemotherapy treatment (5). Therefore, improving the early detection of GC is a critical approach to decrease the mortality of GC. At present, the commonly used diagnostic methods for GC are gastroscopy and gastrointestinal radiography. Moreover, biopsy is the gold standard for pathological confirmation. Their application as a screening test is restricted due to the invasiveness and high cost. Several serum biomarkers, including carcinoembryonic antigen (CEA), carbohydrate antibody 199 (CA199), and carbohydrate antibody 724 (CA724), have been used in clinics to evaluate the effectiveness of therapy (6, 7). However, these serum biomarkers have limited sensitivity and specificity for cancer screening (8, 9). Therefore, it is important to find novel, reliable, and non-invasive blood biomarkers to improve the detection of GC.

With the development of cancer, the abnormal expression of tumor-associated antigen (TAAs) can trigger an autoimmune response, and the corresponding antibodies are called autoantibodies against the tumor-associated antigen (TAAbs) (10–12). Many studies have shown that TAAbs can be detected before the diagnosis of cancer and can stay in the serum longer than tumor-associated antigens (TAAs) (13, 14). TAAs and TAAbs have been reported as potential biomarkers for the early detection of cancers (15–18).

**Figure 1:**
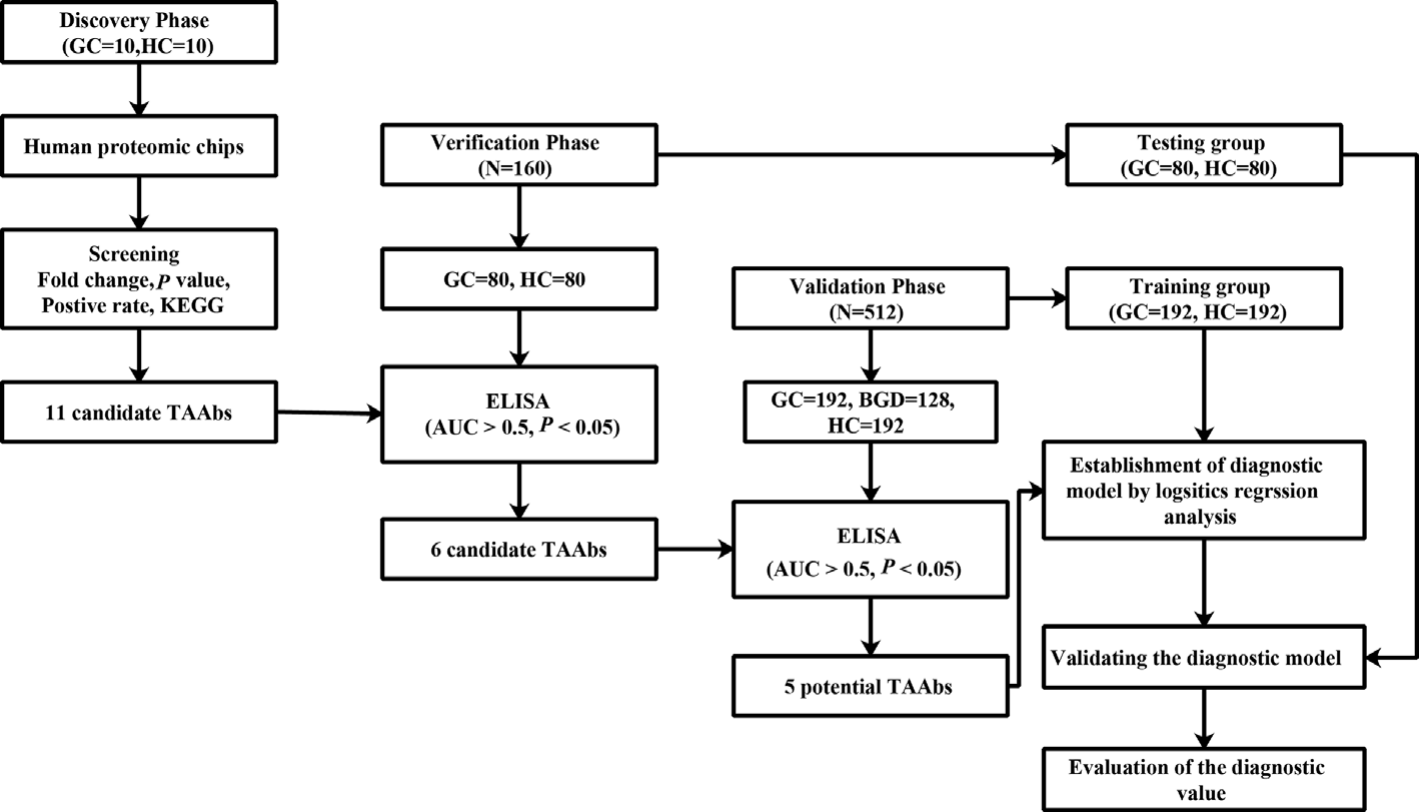
The design of this study. GC, gastric cancer; HCs, healthy controls; ELISA, enzyme-linked immunosorbent assay.

Proteomic chip is a high-throughput technology for cancer biomarker development. It can simultaneously analyze serum autoantibodies against many proteins for further screening and identifying novel TAAbs (19–21). Therefore, in this study, we used the human proteomic chip, which contains more than 21,000 recombinant human proteins, to identify TAAbs to detect GC. The design of the present study is illustrated in **Figure 1**.

## Materials and Methods

### Serum Samples

A total of 692 samples were included in this study, including 282 GC cases, 282 healthy controls (HCs), and 128 benign gastric diseases (BGD) cases. GC and BGD serum samples were collected from a tertiary Level A hospital of Zhengzhou (January 2012 to June 2017). All patients were histopathologically confirmed and had not received any treatments. All HCs were selected from the biobank of Henan Key Laboratory of Tumor Epidemiology and were free of any digestive tract diseases and autoimmune diseases. In the discovery phase, 10 GC cases and 10 HCs were selected, and in the verification and validation phase, 672 subjects were selected, among which the verification cohort included 80 GC cases and 80 HCs, while the validation cohort included 192 GC cases, 192 HCs, and 128 BGD cases. All GC cases and HCs were matched by frequency matching method. The serums were collected according to the standard protocol. This study was approved by the Institutional Review Board of Zhengzhou University and informed consent forms were received from all participants.

### Human Proteomic Chips

In this study, HuProt™ human proteomic chips were purchased from BCBIO Biotechnology (Guangzhou, China). HuProt™ human proteomic chip used in this study is by far the world’s largest collection of full-length human proteins (https://cdi.bio/huprot/) available. It contains more than 21,000 recombinant proteins and covers all human recombinant proteins that can be purified, covering 81% of the human proteome. It is the most comprehensive chip available at present. More detailed information about proteomic chip can be found at https://cdi.bio/huprot/. Theoretically, it is the most comprehensive protein-chip to select possible TAAbs specific to gastric cancer. The human proteomic chips were used to detect the titer of TAAbs in serum samples from 10 GC cases and 10 HCs, to screen candidate TAAbs for GC detection. The experiment was carried out according to the manufacturer’s protocol and their previous publication (22).

### Enzyme-Linked Immunosorbent Assay

The titer of TAAbs was detected in serum samples by indirect enzyme-linked immunosorbent assay (ELISA). The protocol was described in detail in our previous study (23). In this study, a verification cohort was first used to verify the eligibility of candidate TAAbs screened from the proteomic chip, and then the diagnostic value of TAAbs was further validated by the validation cohort. 0.125, 0.25, 0.25, 0.25, 0.25, 0.25, 0.25, 0.25, 0.25, 0.25, and 0.25 µg/ml were the coating concentrations of 11 recombinant human proteins (INPP5A, F8, NRAS, MFGE8, PTP4A1, RRAS2, RGS4, RHOG, SRARP, RAC1, and TMEM243), respectively. A serial of different concentrations of human IgG (Solarbio, China) were used for quality control.

### Statistical Analysis

GenePix Pro 6.0 was used to acquire the original data from proteomic chips. IBM SPSS statistical software (version 21.0), GraphPad Prism 6.0, and MedCalc 11 were used to analyze the data. Nonparametric test was used to analyze the difference of TAAbs level between different groups. The sensitivity, specificity, and AUC were calculated by receiver operating characteristic (ROC) curve analysis. Meanwhile, the positive predictive value (PPV), negative predictive value (NPV), and Yoden index were used to evaluate the validity and reliability of the diagnostic tests using serum autoantibodies as biomarkers. When the specificity was greater than 85%, the maximum Yoden index (YI) was used to set the cutoff value of TAAbs to determine the positive reaction. Logistic regression analysis was used to establish the optimal model to distinguish GC from NC. All *P* values were determined based on two-tailed, and *P*<0.05 was defined to be significant.

## Results

### Characteristics of the Study Population

The experiment was divided into the discovery phase, the verification phase, and the validation phase. In the discovery phase, the titer of TAAbs in serum samples from 10 GC cases and 10 HCs were measured by human proteomic chips. In the verification phase and validation phase, ELISA was applied to test the title of 11 candidate TAAbs in serums from 272 GC cases, 272 HCs, and 128 BGD cases. The clinical characteristics of all participants were shown in **Table 1**. There was no significant difference in clinical characteristics between the two cohorts. All patients were graded according to the TNM staging criteria of the International Union for Cancer Control (UICC).

**Table 1:** Characteristics of all subjects in this study.

Variables	Verification cohort (n=160)		Validation cohort (n=512)		
	GC (n=80)	HC (n=80)	GC (n=192)	BGD (n=128)	HC (n=192)
**Year**					
Range	31–94	35–80	30–83	17–85	35–74
Median (Q1, Q3)	60.6 (53.0–68.0)	59.0 (54.0–62.0)	58.4 (50.0–68.0)	54.0 (46.0–64.0)	56.9 (51.0–65.0)
**Sex, n (%)**					
Male	55 (70.0)	55 (70.0)	136 (71.0)	46 (35.9)	136 (71.0)
Female	25 (30.0)	25 (30.0)	56(29.0)	82 (64.1)	56 (29.0)
**Family cancer history**					
Yes	13 (16.3)		32 (16.7)	27 (21.1)	
No	67 (83.7)		151 (78.6)	86 (67.2)	
Unknown	0 (0)		9 (4.7)	15 (11.7)	
**TNM stage, n (%)**					
Stage I	11 (13.8)		26 (13.5)		
Stage II	12 (15.0)		42 (21.9)		
Stage III	24 (30.0)		56 (29.2)		
Stage IV	4 (5.0)		16 (8.3)		
Unknown	29 (36.2)		52 (27.1)		
**Differentiated degree**					
Poorly	30 (37.5)		73 (38.0)		
Moderately	24 (30.0)		61 (31.8)		
Highly	1 (1.2)		4 (2.1)		
Unknown	25 (31.3)		54 (28.1)		
**Depth of tumor invasion**					
T1	5 (6.2)		8 (4.2)		
T2	8 (10)		19 (9.9)		
T3	10 (12.5)		21 (10.9)		
T4	22 (27.5)		48 (25.0)		
Unknown	35 (43.8)		96 (50.0)		
**Lymph node metastasis**					
Yes	31 (38.8)		67 (34.9)		
No	14 (17.5)		28 (14.6)		
Unknown	35 (43.7)		97 (50.5)		
**Distant metastasis**					
Yes	4 (5)		7 (3.6)		
No	45 (56.3)		95 (49.5)		
Unknown	31 (38.7)		90 (47.4)		

### Candidate TAAbs

Based on the SNRs of 20 serum samples in the proteomic chips, four criteria were used to screen the candidate TAAbs. (1). Mann-Whitney U test was used to compare whether there was a statistical difference in SNR between GC cases and HCs. A *P* value of <0.05 (two sided) was considered to be significant. (2). Fold change (FC) of GC cases against HCs was calculated, and FC ≥ 1.2 was used as a cutoff value to select potential TAAbs. (3). When the difference of positive rate (cut off = mean + standard error) of SNR between GC cases and HCs was more than 80%, the protein was identified as a candidate TAAb. (4). KEGG analysis was performed to select cancer-associated proteins as candidate TAAs. Finally, 11 candidate TAAs were identified, and autoantibodies against these 11 TAAs, including INPP5A, F8, NRAS, MFGE8, PTP4A1, RRAS2, RGS4, RHOG, SRARP, RAC1, and TMEM243 were evaluated as potential markers in GC. The basic characteristics of 11 TAAs were shown in **Table S1**.

### Autoantibodies in Verification Cohort and Validation Cohort

**Figure 2:**
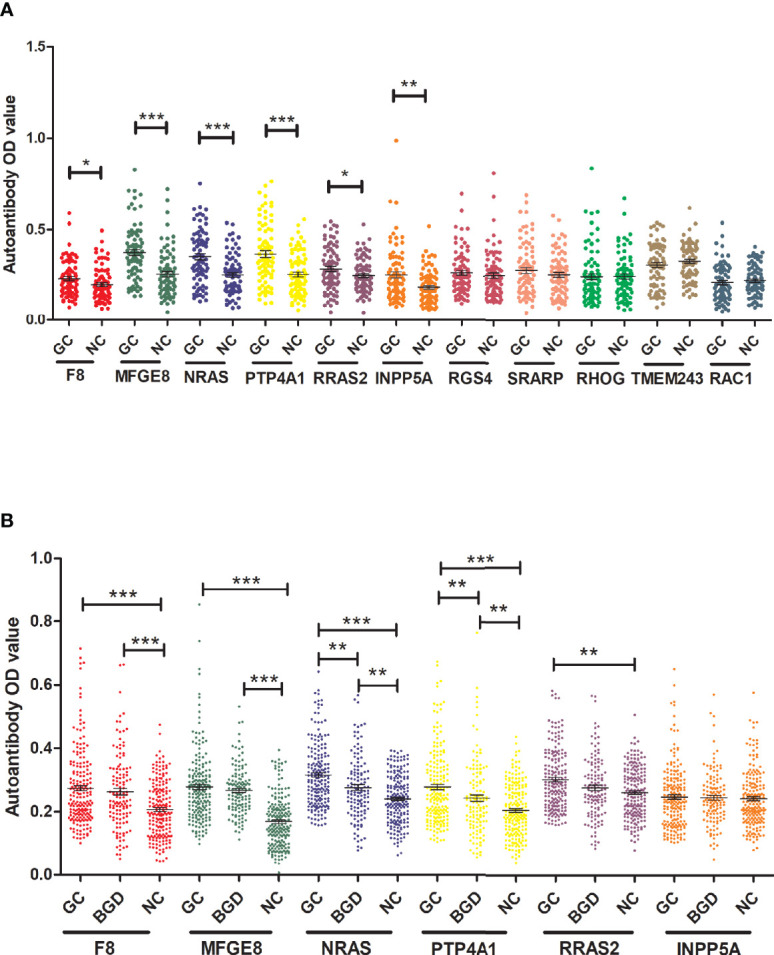
The titer of 11 anti-TAAs autoantibodies in GC cases and HCs. **(A)** Scatter plots of the titer of autoantibodies in the verification cohort, **(B)** scatter plots of the titer of autoantibodies in the validation cohort. GC, gastric cancer; HC, healthy controls; BGD, benign gastric disease. (*P < 0.05, **P < 0.01, ***P < 0.001).

**Figure 3:**
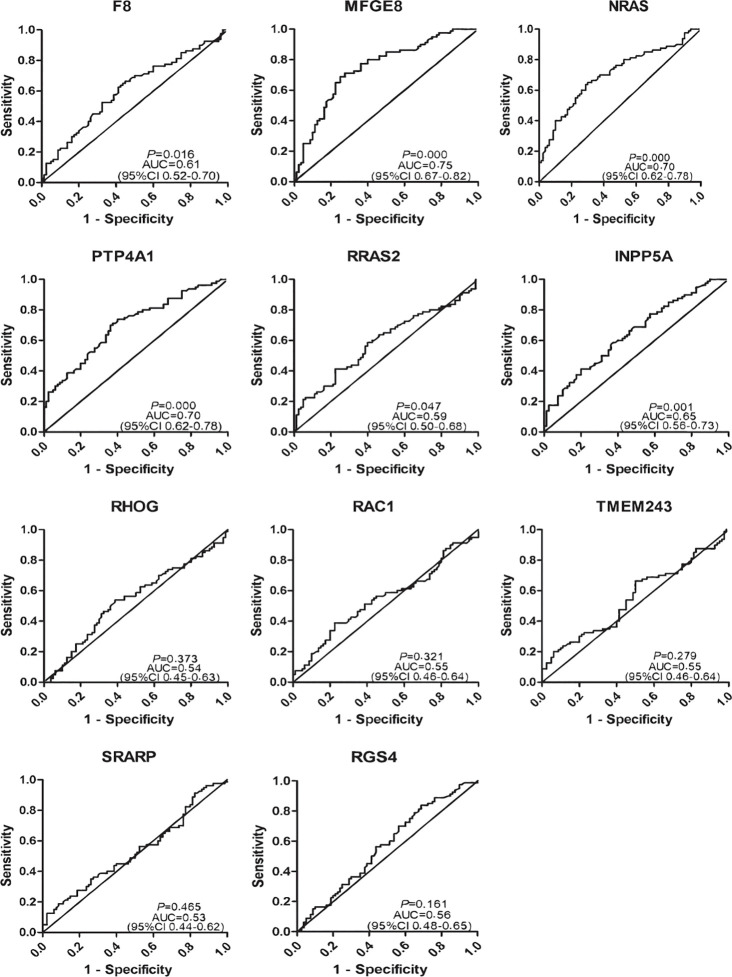
Diagnostic performance of 11 anti-TAAs in the verification cohort for gastric cancer (GC) detection.

To determine the diagnostic value of the 11 aforementioned candidate TAAbs, two independent cohorts were selected to detect their titer. First, in the verification cohort, 160 serum samples were detected by ELISA. The OD values of the 11 TAAbs in the verification cohort were shown in **Figure 2A**. The titer of autoantibodies against INPP5A, F8, NRAS, MFGE8, PTP4A1, and RRAS2 was significantly higher in GC cases while the titer of autoantibodies against RGS4, RHOG, SRARP, RAC1, and TMEM243 showed no difference between these two groups. **Figure 3** showed the ROC curves of the 11 candidate TAAbs. The AUC ranged from 0.53 to 0.75, the sensitivity was 18.8 to 83.8%, and the specificity was 31.3 to 91.3%. Anti-MFGE8 showed the highest diagnostic value with an AUC of 0.75 (95% CI: 0.68–0.82), and the optimal sensitivity and specificity were 71.3 and 72.5%, respectively.

**Figure 4:**
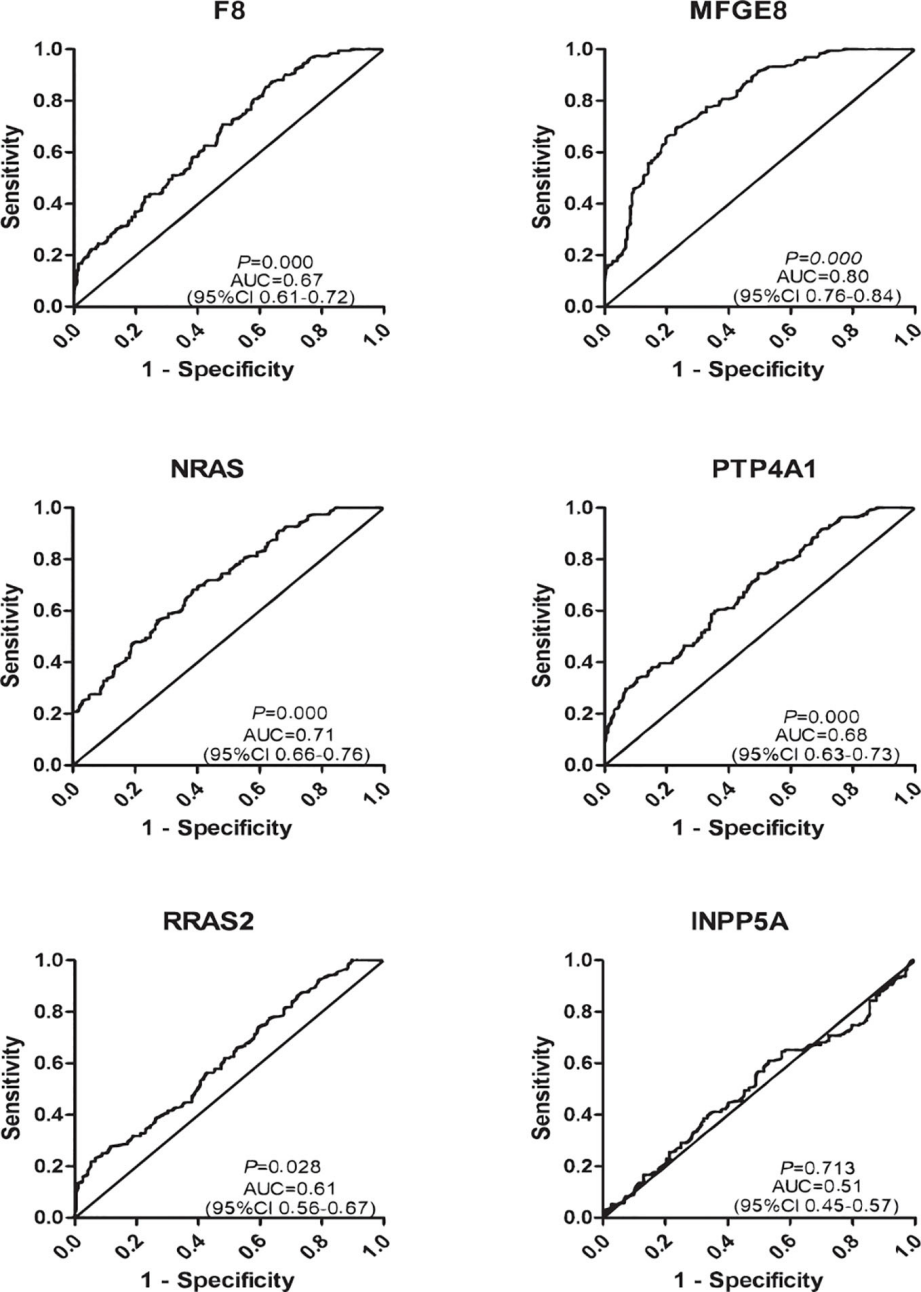
Diagnostic performance of six anti-TAAs in the validation cohort for gastric cancer (GC) detection.

Further, six potential TAAbs were validated by ELISA in the validation cohort, including 192 GC cases, 128 BGD cases, and 192 HCs. **Figure 2B** showed the OD values of the six TAAbs. The titer of autoantibodies against F8, NRAS, MFGE8, RRAS2, and PTP4A1 was significantly higher in GC cases. Besides, the titer of autoantibodies against NRAS and PTP4A1 in GC cases were significantly higher than that in BGD cases. The ROC curves of 6 potential TAAbs were shown in **Figure 4**. The AUCs of six potential TAAbs ranged from 0.51 to 0.80. The sensitivity and specificity ranged from 23.4 to 87.5% and 36.0 to 93.2%, respectively. Among them, anti-MFGE8 showed the best diagnostic value with an AUC of 0.80 (95% CI: 0.76–0.84), the optimal sensitivity and specificity were 69.3 and 77.1%, respectively. The diagnostic value of single TAAbs for GC detection is shown in **Table 2**.

**Table 2:** Diagnostic value of 11 anti-TAAs autoantibodies in verification cohort and validation cohort for gastric cancer (GC) detection.

TAAbs	Verification cohort						Validation cohort					
	Se (%)	Sp (%)	AUC	95% CI	Accuracy (%)	*P*	Se (%)	Sp (%)	AUC	95% CI	Accuracy (%)	*P*
F8	58.8	62.5	0.61	0.52–0.70	60.6	0.016^*^	87.5	36.0	0.67	0.61–0.72	61.7	0.000^***^
MFGE8	71.3	72.5	0.75	0.67–0.82	71.9	0.000^***^	69.3	77.1	0.80	0.76–0.84	73.2	0.000^***^
NRAS	65.0	70.0	0.70	0.62–0.78	66.9	0.000^***^	68.2	61.5	0.71	0.66–0.76	64.8	0.000^***^
PTP4A1	73.8	60.0	0.70	0.62–0.78	66.9	0.000^***^	74.5	50.5	0.68	0.63–0.73	62.5	0.000^***^
RRAS2	58.8	60.0	0.59	0.50–0.68	58.8	0.047^*^	23.4	93.2	0.61	0.56–0.67	58.3	0.028^*^
INPP5A	58.8	62.5	0.65	0.56–0.73	60.6	0.001^**^	60.1	46.9	0.51	0.45–0.57	53.9	0.713
RHOG	53.8	61.3	0.54	0.45–0.63	42.5	0.373						
RAC1	38.8	77.5	0.55	0.46–0.64	41.9	0.321						
TMEM243	66.3	50.0	0.55	0.46–0.64	41.9	0.279						
SRARP	18.8	91.3	0.53	0.44–0.62	55.0	0.465						
RGS4	83.8	31.3	0.56	0.48–0.65	57.5	0.161						

Se, sensitivity, Sp, specificity, AUC, area under the receiver operating characteristic curve; CI, confidence interval. *P < 0.05, **P < 0.01, ***P < 0.001.

### The Establishment of a Diagnostic Model for GC

**Figure 5:**
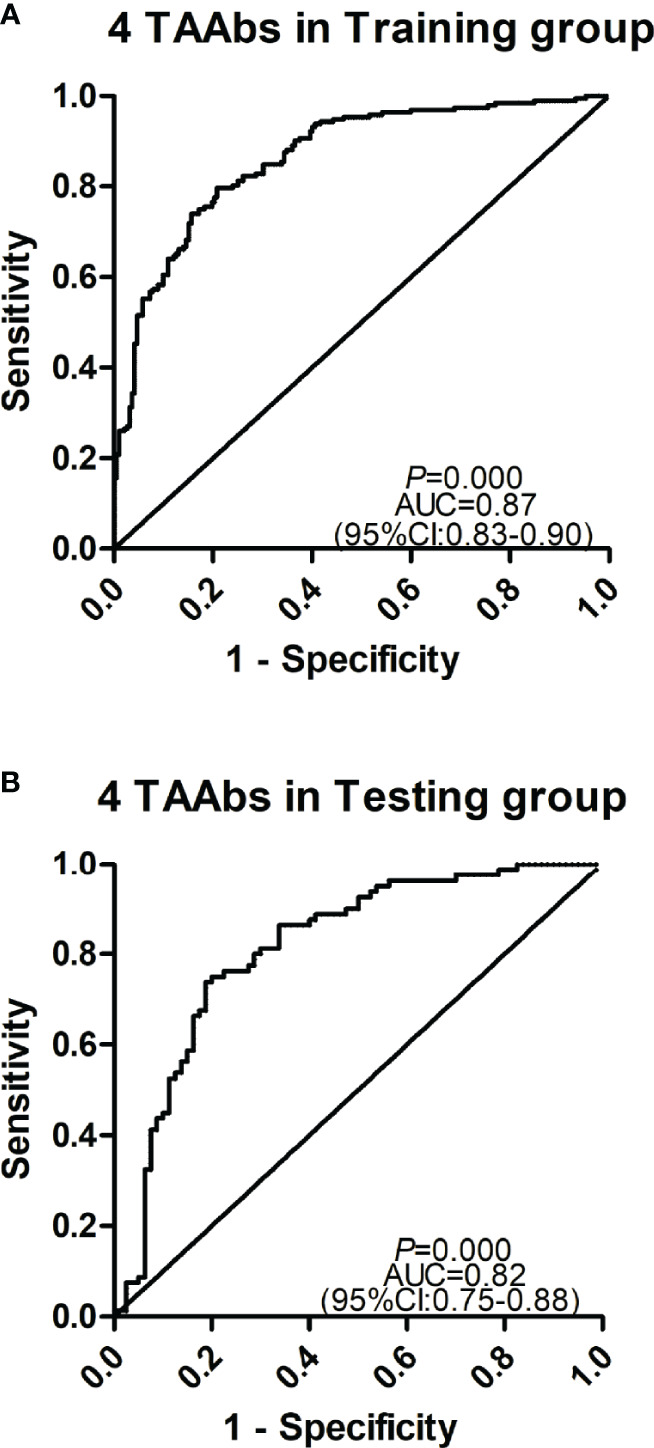
Receiver operating characteristic curve analysis of the prediction model with TAAbs panel in gastric cancer (GC) detection. **(A)** The prediction model with four TAAbs (anti-MFFE8, anti-NRAS, anti-PTP4A1, anti-RRAS2) in the training group. **(B)** The prediction model with four TAAbs in the testing group.

The cohort with a larger sample size was used as the training group to construct a model by logistic regression analysis, and another cohort was used as the testing group to evaluate the model. Based on the OD value of five significant TAAbs (autoantibodies against F8, NRAS, MFGE8, PTP4A1, RRAS2) in the validation cohort (192 GC cases and 192 HCs), logistic regression analysis was used to generate a diagnostic model. The diagnostic model was completely consistent by forward or backward logistic regression methods. Finally, autoantibodies against NRAS, MFGE8, PTP4A1, and RRAS2 entered the model. The predicted possibility for classification as GC was PRE (P = GC, 4 TAAbs) = 1/{1+ EXP [-(−2.517 + 25.928 × anti-NRAS + 14.823 × anti-MFGE8 + 5.862 × anti-PTP4A1 – 32.91 × anti-RRAS2)]}. The AUC of the diagnostic model was 0.87 (95% CI: 0.83–0.90), sensitivity, specificity, and accuracy rates were 70.8, 85.9, and 78.4%, respectively (**Figure 5A**). Then, the verification cohort was used as a testing group to evaluate the diagnostic model. The model obtained from the training group was validated in the testing group (80 GC and 80 NC). The diagnostic value of the model in the testing group was similar to that in the training group, with an AUC of 0.83 (95% CI: 0.76–0.90, *P* < 0.001) (**Figure 5B**). The model is stable since the AUC of the two diagnostic models showed no significant difference (*P* = 0.325).

### Subgroup Analysis of the Diagnostic Model

The GC cases in the training group were divided into different subgroups according to different clinical characteristics and compared with all HCs. The results showed that the model had no significant difference in distinguishing GC cases with different characteristics. The validation group showed similar results (**Table 3**).

**Table 3:** Diagnostic value of the anti-TAAs autoantibodies panel for gastric cancer (GC) patients with different subtype.

Group	n	Se (%)	Sp (%)	Accuracy (%)	AUC	95% CI	*p^a^*	*p^b^*	PPV (%)	NPV (%)	+LR	−LR
Verification cohort												
All patients	80	66.3	85.0	75.6	0.83	0.76–0.90			81.5	71.6	4.4	0.4
≤60	46	76.1	85.0	81.7	0.86	0.80–0.93	0.529	0.212	74.5	86.1	5.1	0.3
>60	34	52.9	85.0	75.4	0.79	0.70–0.88	0.475		60.0	81.0	3.5	0.6
Male	55	67.3	85.0	77.8	0.83	0.76–0.91	1.000	0.858	75.5	79.1	4.5	0.4
Female	25	64.0	85.0	80.0	0.82	0.74–0.91	0.854		57.1	88.3	4.3	0.4
TNM I–II	23	73.9	85.0	82.5	0.85	0.77–0.94	0.716	0.522	58.6	91.9	4.9	0.3
TNM III–IV	28	53.6	86.3	77.8	0.81	0.70–0.89	0.721		57.7	84.1	3.9	0.5
T1–T2	13	76.9	85.0	83.9	0.89	0.83–0.96	0.203	0.252	45.5	95.8	5.1	0.3
T3–T4	32	59.4	86.3	78.6	0.83	0.75–0.91	1.000		63.3	84.1	4.3	0.5
Lymph node(−)	14	85.7	85.0	85.1	0.89	0.86–0.96	0.203	0.257	50.0	97.1	5.7	0.2
Lymph node(+)	31	54.8	86.3	77.5	0.83	0.74–0.91	1.000		60.7	83.1	4.0	0.5
Distant metastasis(−)	45	64.4	85.0	77.6	0.85	0.78–0.92	0.683	0.361	70.7	81.0	4.3	0.4
Distant metastasis(+)	4	50.0	90.0	88.1	0.76	0.58–0.94	0.474		20.0	97.3	5.0	0.6
Poorly differentiated	30	60.0	85.0	78.2	0.80	0.74–0.90	0.853	0.742	60.0	85.0	4.0	0.5
Moderately and highly differentiated	25	68.0	86.3	81.9	0.84	0.76–0.93	0.856		60.7	89.6	4.9	0.4
Validation cohort												
All patients	192	70.8	85.9	78.4	0.87	0.83–0.90			83.4	74.6	5.0	0.3
≥60	98	70.4	85.9	80.7	0.87	0.82–0.91	0.942	0.918	71.9	85.1	5.0	0.3
<60	94	72.3	85.4	81.1	0.87	0.83–0.91	0.967		70.8	86.3	5.0	0.3
Male	136	68.4	86.5	79.0	0.86	0.81–0.90	0.629	0.161	78.2	79.4	5.1	0.4
Female	56	76.8	85.9	83.9	0.90	0.85–0.95	0.301		61.4	92.7	5.5	0.3
TNMI–II	68	67.7	85.4	80.8	0.84	0.79–0.90	0.457	0.590	62.2	88.2	4.6	0.4
TNMIII–IV	72	68.1	85.9	81.1	0.86	0.82–0.91	0.871		64.5	87.8	4.8	0.4
T1–T2	27	77.8	85.4	84.5	0.84	0.75–0.93	0.531	0.535	42.9	96.5	5.3	0.3
T3–T4	69	67.2	87.5	82.1	0.87	0.82–0.92	0.964		65.9	88.1	5.4	0.4
Lymph node(−)	28	75.0	89.6	87.7	0.90	0.84–0.95	0.383	0.187	51.2	96.1	7.2	0.3
Lymph node(+)	67	65.7	86.5	81.1	0.85	0.79–0.90	0.487		62.9	87.8	4.9	0.4
Distant metastasis(−)	95	68.4	87.5	81.2	0.86	0.82–0.91	0.787	0.386	73.0	84.8	5.5	0.4
Distant metastasis(+)	7	85.7	85.9	85.9	0.90	0.82–0.98	0.465		18.2	99.4	6.1	0.2
Poorly differentiated	73	74.0	85.4	82.3	0.85	0.80–0.91	0.627	0.346	65.9	89.6	5.1	0.3
Moderately and highly differentiated	65	66.2	89.1	83.3	0.89	0.85–0.93	0.547		67.2	88.6	6.0	0.4

Se, sensitivity; Sp, specificity; AUC, area under the receiver operating characteristic curve; CI, confidence interval; PPV, positive predictive value; NPV, negative predictive value; +LR, positive likelihood ration; −LR, negative likelihood ration.

P^a^ means comparison between TNM stage or T stage or lymph node or distant metastasis or differentiated and all patients with the method of De Long et al. (24).

P^b^ means comparison between ≥60 and <60 or TNMI–II and TNMIII–IV stage or T1–T2 and T3–T4 or lymph node (−) and lymph node (+) or distant metastasis (−) and distant metastasis (+) or poorly differentiated and moderately and highly differentiate.

### The Specificity of Four TAAbs in Detecting GC

**Figure 6:**
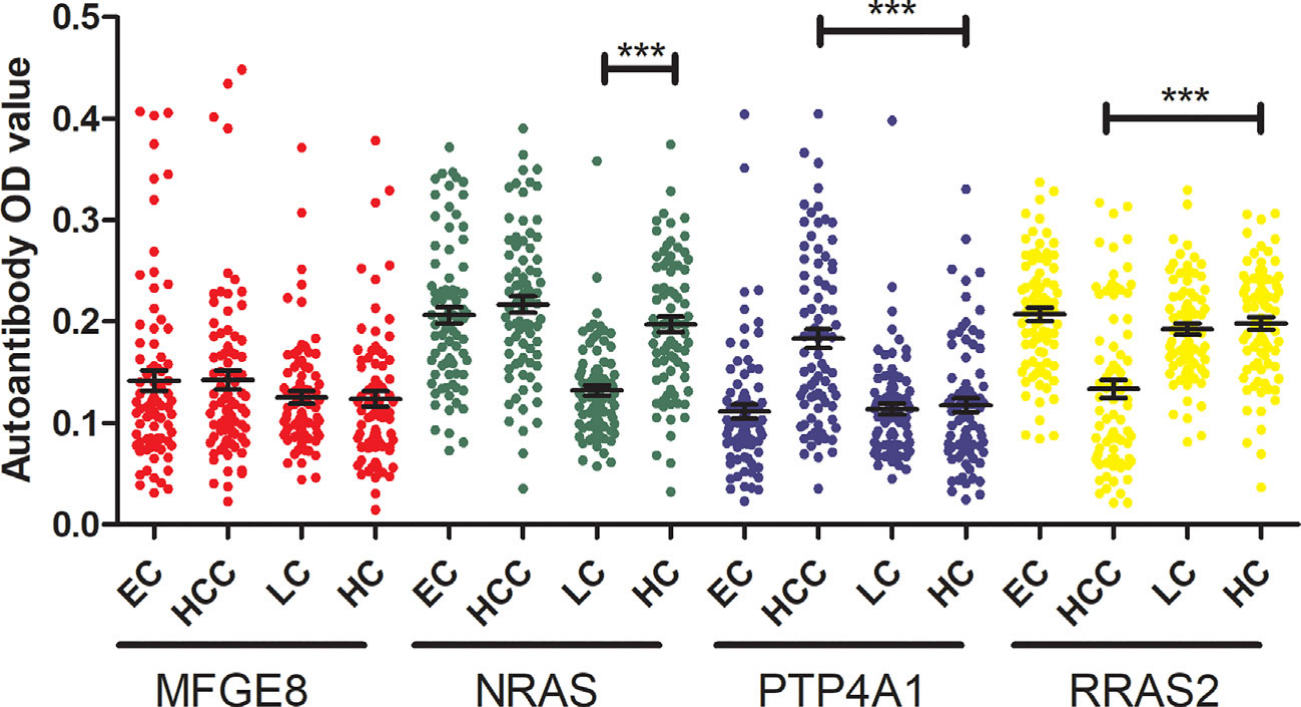
The titer of four TAAbs in other types of cancer and HCs. (***P < 0.001).

To verify the specificity of four TAAbs in gastric cancer. The titer of autoantibodies against MFGE8, NRAS, PTP4A1, RRAS2 was measured in serum of 80 esophagus cancer cases (ECs), 80 hepatocellular carcinoma cases (HCCs), 80 lung cancer cases (LCs), and 80 healthy controls by ELISA. The OD values of four TAAbs were shown in **Figure 6**. Only the titer of anti-PTP4A1 in HCCs was higher than that in healthy controls. Moreover, the titer of anti-NRAS in healthy controls was higher than that in LCs, anti-RRAS2 in healthy controls was higher than that in HCCs.

### Establishment of a Diagnostic Model for BGD

To explore the progression pattern of healthy cases to BGD cases to GC cases, we compared the diagnostic value of single TAAb for BGD cases and established a diagnostic model. Based on the results of ELISA in the validation cohort (128 BGD cases, 192 HCs), the titer of four TAAbs (autoantibodies against F8, MFGE8, NRAS, PTP4A1) were significantly higher in BGD cases (**Figure 2B**). The ROC curves of six TAAbs were shown in **Figure S1**. Logistic regression analysis was used to generate a diagnostic model. Finally, autoantibodies against NRAS and MFGE8 entered the model. The AUC of the diagnostic model was 0.84 (95% CI: 0.79–0.88), sensitivity, specificity, and accuracy rates were 61.7, 86.5, and 71.6%, respectively (**Figure S2**).

## Discussion

GC ranked the fifth of most common cancer and the third leading causes of cancer death worldwide (1). At present, gastroscopy and gastrointestinal radiography are the most common diagnostic techniques of GC. However, for early-stage GC, these procedures do not show a satisfying diagnostic value. Due to the early-stage GC are asymptomatic, most patients were late-stage when they were diagnosed (25). Therefore, it is important to identify a non-invasive diagnostic method for GC. Many studies have reported that TAAbs could be stable in serum and be considered a potential biomarker for cancer detection (26–28).

In this study, 11 candidate TAAbs for diagnostic GC were identified by human proteomic chips in 20 serum samples, and the titer of 11 TAAbs in 672 samples was detected by ELISA. The diagnostic model for GC cases was established, and autoantibodies against NRAS, MFGE8, PTP4A1, and RRAS2 entered the model. The AUC of the diagnostic model was 0.87 (95% CI: 0.83–0.90), sensitivity, specificity, and accuracy rates were 70.8, 85.9, and 78.4%, respectively, in the training group. The results indicated that the model was stable. Although many studies have reported that optimal combinations of autoantibodies could aid in the diagnosis of GC, the results from the current study showed better performance in distinguishing GC and HC.

Several approaches have been used to identify valuable TAAbs in cancers, among which serological analysis of expression cDNA libraries (SEREX) and serological proteome analysis (SERPA) are the most commonly used technologies (29–31). However, the false-positive rate of SEREX is too high, and the construction of a cDNA gene expression library from patients is time-consuming and unrepresentative. Moreover, SEREX cannot screen post-translational modified protein (32, 33). SERPA can only screen relatively high levels of proteins and consumes a large portion of serum (31). In recent years, with the development of proteomic chips, which can be screen TAAbs in a high throughput way, have been used by more and more researchers to screen TAAbs (31, 34). One recent study used human proteomic chips to detect TAAbs in GC and HCs to discover candidate biomarkers (35).

Based on the human proteomic chips, 11 candidate TAAbs (autoantibodies against INPP5A, F8, NRAS, MFGE8, PTP4A1, RRAS2, RGS4, RHOG, SRARP, RAC1, and TMEM243) were identified. Then, two independent cohorts were used to further identify TAAbs. Finally, five potential TAAbs (autoantibodies against F8, NRAS, MFGE8, PTP4A1, RRAS2) were identified by ELISA. The five potential biomarkers have not been reported in GC. F8 belongs to the coagulation factor family and plays a vital role in the coagulation cascade (36). Some studies have shown that the expression of F8 is high in multiple myeloma, breast cancer, and colorectal cancer (37–40). NRAS is a GDP binding gene, an important component of the RAS pathway associated with many cancers (41, 42). MFGE8 is a secreted glycoprotein protein and closely related to immune tolerance and homeostasis by promoting phagocytosis of apoptotic cells (43, 44). As a biomarker, MFGE8 has already been reported in breast cancer (45). PTP4A1 can enhance cell proliferation, cell motility, and invasive activity and promote cancer metastasis (46, 47). RRAS2 is a member of the Ras-related subfamily, with GTPase activity involved in regulating the MAPK signaling pathway, thereby controlling multiple cellular processes (48).

Many studies have shown that TAAbs can be used to detect cancer. However, previous studies also have shown that the diagnostic performance of a single anti-TAA autoantibody was not sufficient to be used in the screening of cancers (15, 49). In our study, two independent cohorts (verification cohort and validation cohort) were used to verify the discovered TAAbs and evaluate the diagnostic value of a single TAAb for GC cases. Finally, five potential TAAbs (autoantibodies against F8, NRAS, MFGE8, PTP4A1, RRAS2) were identified. The ranges of AUC, sensitivity, specificity was 0.51–0.80, 23.4–87.5, and 36.0–93.2%, respectively. These results are consistent with previous reports (15, 49). Meanwhile, many studies have shown that combinational utilization of multiple TAAbs may potentially improve the diagnostic accuracy for cancers. A recent study showed that a panel of four TAAs (COPS2, CTSF, NT5E, and TERF1) can effectively diagnose GC with 95% sensitivity and 92% specificity. But the results were not further validated (50). Hideaki et al. revealed that an array of six TAAs (p53, heat shock protein 70, HCC-22-5, peroxiredoxin VI, KM-HN-1, and p90) was capable of discriminating GC cases from HCs with sensitivity/specificity of 49.0/92.4% and 52.0/90.5% in test cohort and validation cohort (51). Another study reported that an array with a 45-autoantibody signature could distinguish GC patients from HCs, with an AUC of 0.79, the sensitivity of 58.7%, and specificity of 89.7% in the validation set (52). However, these two studies did not construct a prediction model of GC and, therefore, they did not show which TAA was more closely related to the occurrence of GC. Logistic regression analysis is one conventional statistical method that has been widely adopted to classify cancers. In our study, the diagnostic model was established by logistic regression analysis, then autoantibodies against NRAS, MFGE8, PTP4A1, and RRAS2 entered the model (AUC = 0.87, sensitivity = 70.8%, and specificity = 85.9%). And the diagnostic value of this panel was confirmed in the testing group.

In addition, we selected the serum of 80 ECs, 80 HCCs, and 80 LCs and measured the titer of four TAAbs (anti-MFGE8, anti-NRAS, anti-PTP4A1, anti-RRAS2) by ELISA to verify the specificity of four TAAbs in gastric cancer. The results showed that the titer of anti-PTP4A1 in HCCs was higher than that in healthy controls. Many studies shown that PTP4A1 is highly correlated with the occurrence, development, and prognosis of HCC (53, 54). Moreover, the results showed that the titer of anti-NRAS in healthy controls was higher than that in LCs, anti-RRAS2 in healthy controls was higher than that in HCCs. However, TAAbs are produced by immune response and have amplification effect and TAAbs with higher levels are more likely to be detected in cancer patients. Therefore, the levels of anti-NRAS in LCs and anti-RRAS2 in HCCs are still very low after amplification effect, which may not be good diagnostic markers.

There are some advantages in the current study. Firstly, the human proteomic chips were used to screen candidate TAAbs associated with GC and had yielded promising results. Secondly, the diagnostic model was established by logistic regression analysis in the training group, and another independent group was used to test this model’s performance. However, some limitations also need to be mentioned. Firstly, all proteins on the proteomic chips were homogeneously expressed from normal human coding genes, so it is hard to identify the TAAbs with structural changes and post-translational modification aberrance. Secondly, further validations are warranted to confirm the results from the current study.

In summary, the proteomic chip approach is a feasible method to identify TAAbs for the detection of cancer. Moreover, the diagnostic panel (anti-NRAS, anti-MFGE8, anti-PTP4A1, anti-RRAS2) may be useful to distinguish GC cases from HCs.

## Data Availability Statement

The original contributions presented in the study are included in the article/**Supplementary Material**. Further inquiries can be directed to the corresponding authors.

## Ethics Statement

The studies involving human participants were reviewed and approved by The Institution Review Board of Zhengzhou University. The patients/participants provided their written informed consent to participate in this study.

## Author Contributions

JZ and JS conceived the design of the current study. CC and YD conducted experiments and drafted the manuscript. CQ, GS, and ZH participated in the data analysis. PW, HY, KW, LD, and CS helped to draft the manuscript. All authors contributed to the article and approved the submitted version.

## Funding

This work was supported by the Major Project of Science and Technology in Henan Province (No.161100311400), the Zhengzhou Major Project for Collaborative Innovation (No.18XTZX12007), the National Science and Technology Major Project of China (2018ZX10302205), and the Project of Basic Research Fund of Henan Institute of Medical and Pharmacological Sciences (2019BP0202).

## Conflict of Interest

The authors declare that the research was conducted in the absence of any commercial or financial relationships that could be construed as a potential conflict of interest.
