# Supplementary material for: Identification of Novel Autoantibodies Based on the Human Proteomic Chips and Evaluation of Their Performance in the Detection of Gastric Cancer
Source: Front Oncol. 2021 Feb 26;11:637871. doi: 10.3389/fonc.2021.637871 (PMC7953047; doi:10.3389/fonc.2021.637871)
Supplement: Supplementary file 1 [file DataSheet_1.docx]

Supplementary Material

## Supplementary Figure 1


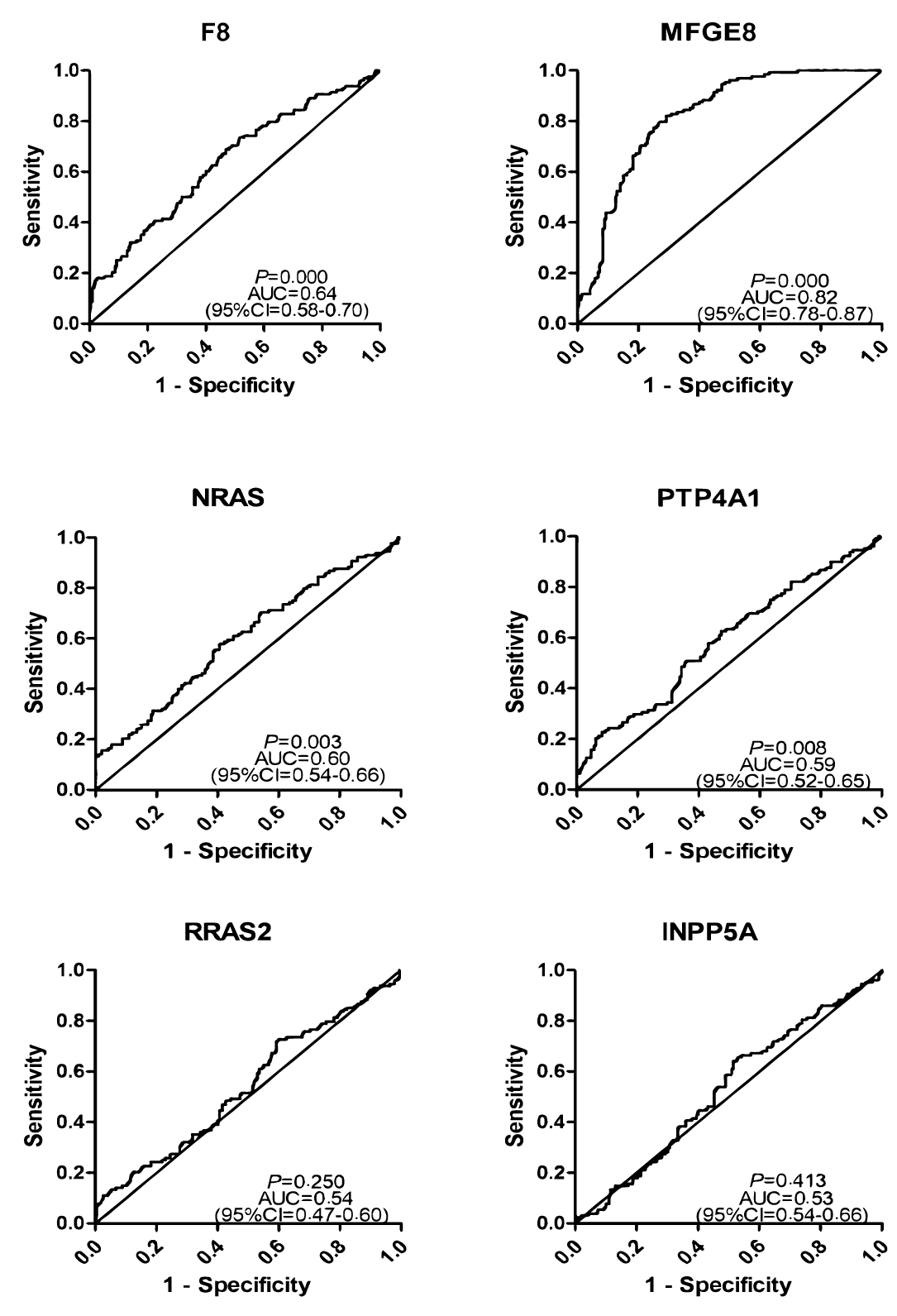


Supplementary Figure 1. Diagnostic performance of 6 anti-TAAs in the verification cohort for BGD detection.

## Supplementary Figure 2


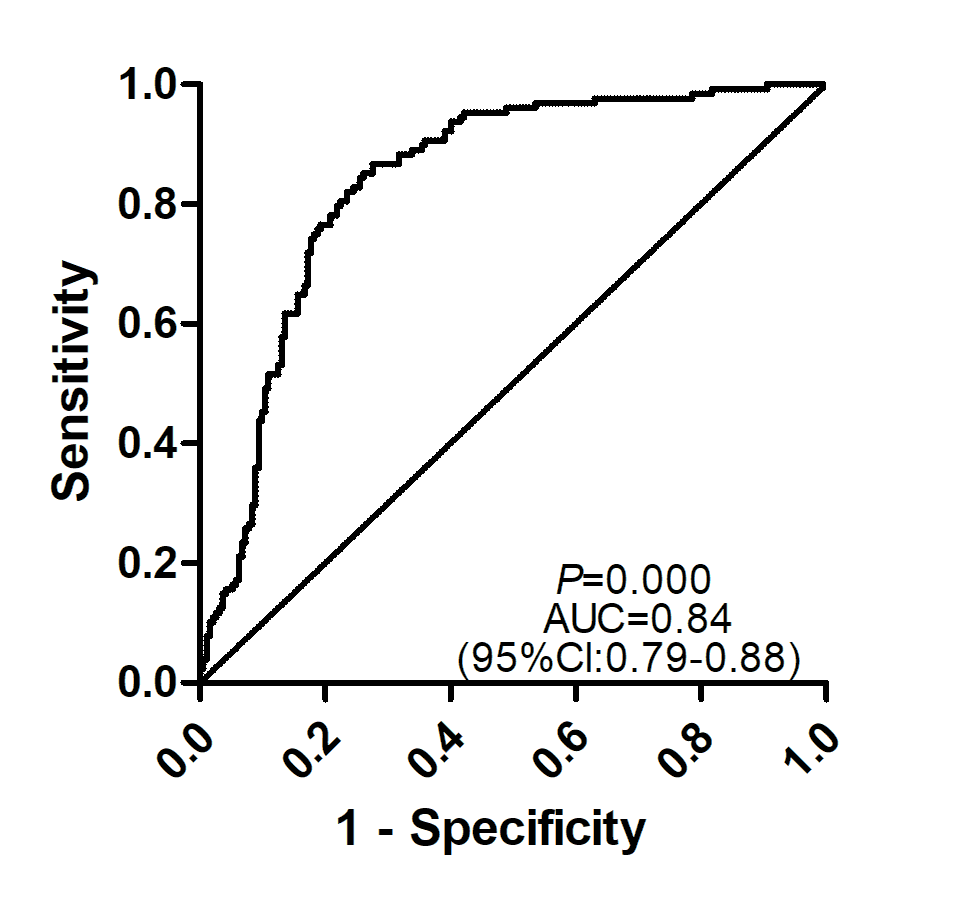


Supplementary Figure 2. Receiver operating characteristic curve analysis of the prediction model with 2 TAAbs (anti-MFGE8, anti-NRAS) panel in BGD detection.

## Supplementary Table 1

**TableS1** Basic characteristics of screened candidate TAAs

| TAAs | Coded genes | PositiveGC (%) | PositiveHC (%) | FC | P |
| --- | --- | --- | --- | --- | --- |
| F8 | Coagulation factor VIII | 80.0 | 0.0 | 1.67 | 0.000 |
| MFGE8 | Lactadherin | 80.0 | 0.0 | 1.84 | 0.000 |
| NRAS | GTPase NRas | 80.0 | 0.0 | 1.86 | 0.000 |
| PTP4A1 | Protein tyrosine phosphatase type IVA 1 | 90.0 | 0.0 | 2.01 | 0.000 |
| RRAS2 | Ras-related protein R-Ras2 | 90.0 | 0.0 | 2.19 | 0.000 |
| INPP5A | Inositol polyphosphate-5-phosphatase A | 90.0 | 0.0 | 1.78 | 0.000 |
| RHOG | Rho-related GTP-binding protein RhoG | 90.0 | 0.0 | 1.93 | 0.000 |
| RAC1 | Ras-related C3 botulinum toxin substrate 1 | 90.0 | 0.0 | 2.16 | 0.000 |
| TMEM243 | Transmembrane protein 243 | 80.0 | 0.0 | 1.86 | 0.000 |
| SRARP | Steroid receptor-associated and regulated protein | 80.0 | 0.0 | 1.62 | 0.000 |
| RGS4 | Regulator of G-protein signaling 4 | 90.0 | 10.0 | 1.68 | 0.000 |
